# Mendelian susceptibility to mycobacterial disease: Clinical, immunological, and genetic study in India

**DOI:** 10.70962/jhi.20250153

**Published:** 2025-12-18

**Authors:** Aparna Dalvi, Lavina Temkar, Mukesh Desai, Swati Garg, Jahnavi Aluri, Gouri Hule, Umair Ahmed Bargir, Priyanka Setia, Priyanka Kambli, Amruta Dhawale, Parag Tamhankar, Reetika Malik Yadav, Sagar Bhattad, Meena Sivasankaran, Harsha Prasad Lashkari, Asruti Kacha, T. Kasi Bharathi, Swati Kanakia, Kamana Arun Gandhi, Shraddha Shelar, Shweta Shinde, Neha Jodhawat, Ramesh Kawale, Nitin Salve, Jean-Laurent Casanova, Jacinta Bustamante, Manisha R. Madkaikar

**Affiliations:** 1 Indian Council of Medical Research National Institute of Immunohaematology, Parel, India; 2 St. Giles Laboratory of Human Genetics of Infectious Diseases, Rockefeller University, New York, NY, USA; 3 Division of Immunology, Bai Jerbai Wadia Hospital for Children, Mumbai, India; 4 Centre for Medical Genetics, Mulund, India; 5 Foundation for Primary Immunodeficiency Diseases Centre of Excellence for Diagnosis and Management of Primary Immune Deficiencies at Aster Group of Hospitals, Bangalore, India; 6 Department of Pediatric Hemato-Oncology, Kanchi Kamakoti Childs Trust Hospital, Chennai, India; 7 Kasturba Medical College, Mangalore, India; 8 Manipal Academy of Higher Education, Manipal, India; 9 Department of Pediatrics, Government Medical College, Vadodara, India; 10 Viswabharati Medical College, General and Cancer Hospital, Kurnool, India; 11 Lilawati Hospital and Research Centre, Mumbai, India; 12 Fellowship Course in Pediatric Infectious Diseases, Gujarat, India; 13 University of Paris Cité, Paris, France; 14 Laboratory of Human Genetics of Infectious Diseases, Necker Branch, INSERM UMR1163, Paris, France; 15 Imagine Institute, Necker Hospital for Sick Children, Paris, France; 16 Department of Pediatrics, Necker Hospital for Sick Children, Assistance Publique-Hôpitaux de Paris, Paris, France; 17 Center for the Study of Primary Immunodeficiencies, Necker Hospital for Sick Children, Assistance Publique-Hôpitaux de Paris, Paris, France

## Abstract

Mendelian susceptibility to mycobacterial disease (MSMD) is a rare syndrome characterized by increased and selective susceptibility to weakly virulent mycobacteria and other intramacrophagic pathogens. This study emphasizes the utility of immunological and functional assays in diagnosing MSMD by analyzing clinical, immunological, and genetic features in 50 Indian patients. Immunological workup included lymphocyte subset analysis, nitroblue tetrazolium test (NBT), and flow cytometric assessment of IFN-γR1 (CD119), IL-12Rβ1 (CD212), and phosphorylated STAT1/STAT4 following cytokine stimulation. Functional assays measured IFN-γ and IL-12p70 production. Genetic evaluation was performed using whole-exome or Sanger sequencing. The median age at onset was 3 mo. BCG complications were the most common presentation (96%), while 4% had non-tuberculous mycobacterial infections. Additional infections included *Mycobacterium tuberculosis*, *Salmonella *spp., *Candida *spp., and multiple types of viruses. IL-12Rβ1 deficiency was the most frequent diagnosis, with 10 novel variants in the *IL12RB1* gene identified. These results demonstrate that combining flow cytometry with functional and genetic analyses enables accurate and timely MSMD diagnosis.

## Teaser

Dalvi et al. report on 50 Indian patients with Mendelian susceptibility to mycobacterial disease (MSMD), highlighting its clinical diversity and genetic heterogeneity. The study demonstrates how immunological assays and flow cytometry, combined with genetic testing, enable timely and accurate diagnosis of MSMD, aiding targeted clinical management.

## Introduction

Mendelian susceptibility to mycobacterial disease (MSMD) is a rare yet significant group of inherited disorders marked by heightened vulnerability to severe infections caused by mycobacteria, including nontuberculous mycobacteria, *Mycobacterium tuberculosis *(*MTB*), and other intracellular pathogens (1, 2, 3). These disorders typically arise from genetic variants that impair the immune system’s ability to detect and combat mycobacterial infections, leading to recurrent or severe disease episodes. Key genes involved in MSMD are primarily linked to the interferon-γ (IFN-γ)–mediated immunity, including *IFNGR1*, *IFNGR2*, *IFNG*, *IL12RB1*, *IL12RB2*, *IL23R*, *IL12B*, *ISG15*, *USP18*, *TBX21*, *STAT1*, *TYK2*, *IRF8*, *CYBB*, *IKBKG*, *ZNFX1*, *CCR2*, *JAK1*, *RORC*, *SPPL2A*, *MCTS1*, and *IRF1*, which all play critical roles in immune defense against mycobacterial and other intracellular pathogens (4, 5, 6, 7, 8, 9). The molecular diagnosis of MSMD is complex due to the involvement of multiple genes with both recessive and dominant inheritance patterns. Defects can be either complete or partial, depending on protein expression. Hematopoietic stem cell transplantation (HSCT) is the only cure for complete MSMD, while partial forms can be treated with recombinant IFN-γ alongside antimicrobials. Accurate genetic diagnosis and differentiation between complete and partial defects are essential for the management of these patients.

Various immunological approaches for the molecular and cellular diagnosis of MSMD have been published, facilitating targeted gene sequencing and the prediction of therapeutic responses, such as the effectiveness of adjuvant therapies like exogenous human recombinant IFN-γ (hrIFN-γ) (10). In this paper, we focus on the use of flow cytometric and ELISA-based functional assays for evaluating MSMD patients, aiming to enhance our understanding of the clinical, immunological, and molecular spectrum of MSMD in India.

## Results

### Demographic findings of 50 MSMD patients in India

Between 2014 and 2024, 482 patients with suspected MSMD were referred to our center from various hospitals across India. A total of 50 genetically confirmed cases of MSMD from 44 families were included in the study, some of which had already been published (10). The median age of onset of symptoms was 3 mo (range 2.4–42 mo), and the median age of diagnosis was 17 mo (range 1–180 mo). Of 50, 58% of patients were males, and females were 42%. Consanguinity was seen in 28 families. A family history of a sibling affected was found only in nine families. At the time of follow-up, 20 patients were deceased, 24 remained alive, and six could not be traced due to loss to follow-up.

### Molecular and genetic findings in 50 MSMD

Of the 50 patients studied, molecular characterization of some patients has been previously reported (11). In the remaining patients, next-generation sequencing (NGS) identified 10 novel and 10 previously reported variants across four genes: *IL12RB1*, *IFNGR1*, *IFNGR2*, and *STAT1*. Biallelic variants in *IL12RB1* were found in 20 patients (80%), in *IFNGR1* in three patients (12%), and in *IFNGR2* and *STAT1* in one patient each (4% each; Table S1).

**Figure 1.**
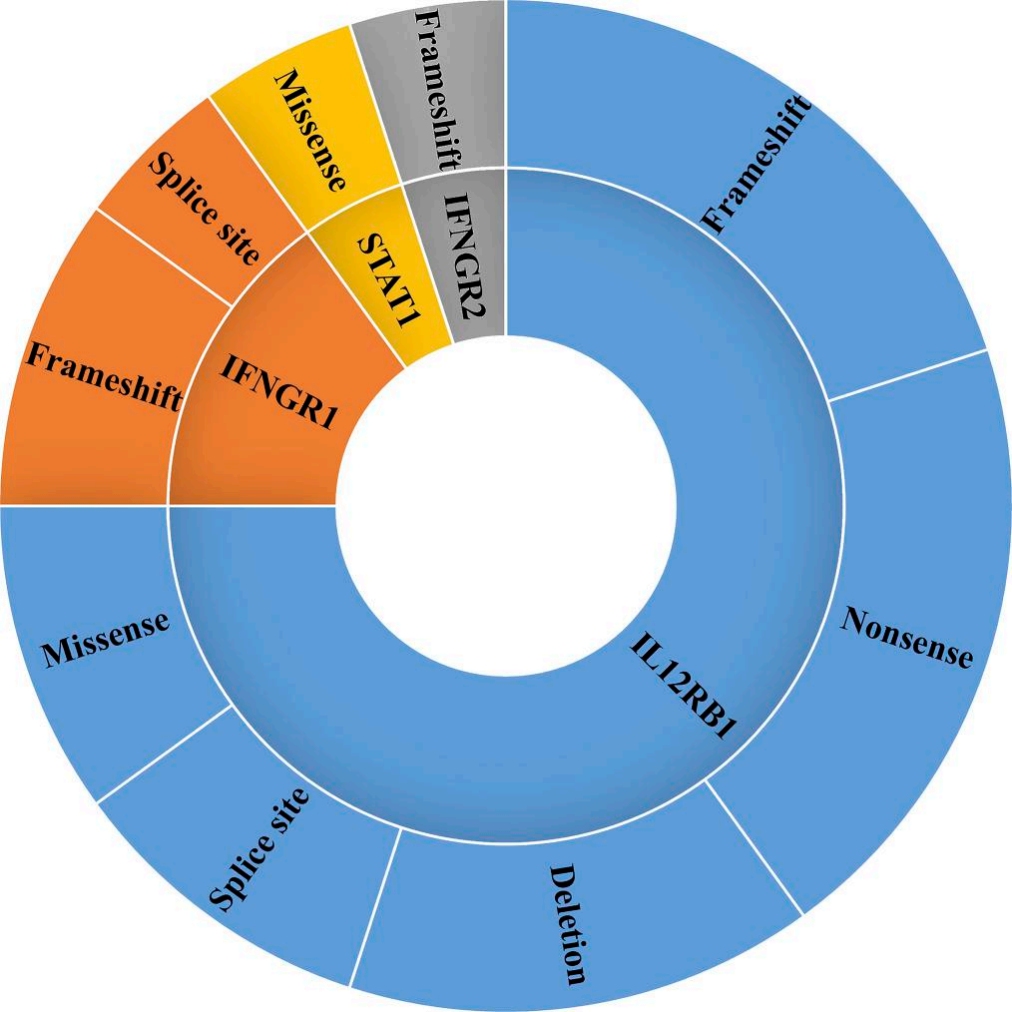
Different types of variants identified in MSMD cohort.

The identified variants comprised frameshift (28%, *n* = 7), nonsense (16%, *n* = 4), missense (12%, *n* = 3), and large deletions (8%, *n* = 2), a small 11–base pair (bp) deletion (4%, *n* = 1), and essential splice-site variants (16%, *n* = 4; Fig. 1). Previously reported *IL12RB1* variants included c.962C>A, c.599del, c.1791+2T>G, c.493C>T, c.632G>C (R211P), c.995G>A, c.494del, c.587G>T (C196F), and c.982del/c.982del. Novel variants identified were c.1242C>G, c.515dup, c.1189+1del, c. (1327+1_1328-1) (1483+1_1484-1) del, c.1095_1106del, and chr19. (18083492_18086759) (18086933_18098745) del. Parental segregation analysis in six families confirmed heterozygosity in both parents. In patient P11, trio-based whole-exome sequencing revealed a large homozygous deletion of exon 12, undetected in either parent, suggesting a possible de novo event.

Three patients harbored pathogenic, homozygous variants in *IFNGR1*. Patient F29_P29, previously described without molecular confirmation, and patient F32_P32 had frameshift variants (c.1068del and c.989_990del, respectively), while patient F31_P31 had an essential splice-site variant (c.85+1G>T). Two of these variants were novel.

In patient F41_P41, a homozygous 8-bp deletion in exon 5 of *IFNGR2* (c.697_704del) was identified, with both parents as heterozygous carriers; this variant was novel. Patient F43_P43 carried a homozygous, private missense variant (c.1553A>C) in exon 18 of *STAT1* (Combined Annotation Dependent Depletion Phred score: 29.4). The affected residue is located in the linker domain between the DNA-binding and SH2 domains. Both parents were heterozygous for this variant.

Variant reclassification was performed in the context of additional information according to the guidelines of the American College of Medical Genetics and Genomics (ACMG). 27 variants were classified as pathogenic. Among the four variants initially classified as variants of uncertain significance (VUS), one was reclassified as likely pathogenic based on accumulated evidence, including supportive computational data, segregation analysis, and a phenotype highly specific for the associated disease, fulfilling the PP4 criterion. The remaining three variants continued to be classified as VUS, despite strong phenotypic correlation, due to the lack of sufficient variant-specific functional evidence to meet other ACMG criteria.

### General clinical findings in 50 MSMD patients

**Figure 2.**
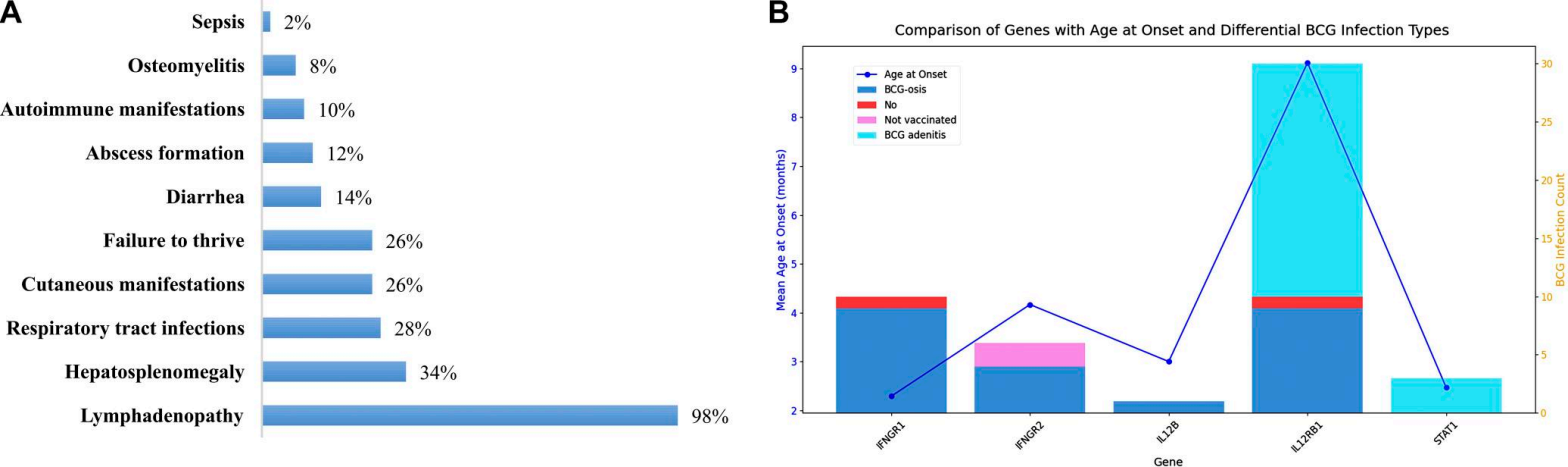
**Bar diagram depicting the distribution of clinical manifestations in MSMD patients and comparison of age at onset and BCG infection types across different MSMD genetic groups. (A)** Clinical findings of MSMD patients. **(B)** Comparison of age at disease onset and BCG infection across different MSMD patient groups.

48 out of 50 patients received the Danish-1331 Bacillus Calmette-Guérin (BCG) vaccine at birth. BCG-related complications were the most common infectious manifestation, observed in 96% of patients. Localized BCG infection (BCG adenitis or BCGitis) occurred in 23 patients (48%), typically involving the left axillary lymph nodes, ipsilateral to the vaccination site. Disseminated BCG infection (BCGosis) was identified in 23 patients (48%), with involvement of the liver, spleen, lungs, skin, bone, and soft tissues. Lymphadenopathy was noted in nearly all affected individuals. Detailed clinical data are summarized in Fig. 2.

### Clinical and microbiological features of patients with impaired IFN-γ production

A total of 30 patients in our cohort had IL-12Rβ1 deficiency, and one had IL-12p40 deficiency. The median age at presentation was 4 mo. Among these patients, 20 (65%) presented with localized BCG adenitis, and 10 (32%) developed BCGosis. Left axillary lymphadenopathy, ipsilateral to the BCG vaccination site, was noted in nearly all patients. Five patients (16%) had both axillary and cervical lymphadenopathy, while six (19%) exhibited multisite lymphadenopathy (cervical, inguinal, mediastinal, perihilar, and abdominal, Table 1).

**Table 1.** Clinical and microbiological findings in patients with impaired IFN-γ production

Patient	Gene	Nucleotide change	Sex	Age at onset	Current age	Consanguinity	BCG infection	Other organ involvement	Failure to thrive	Diarrhea	Other organism	Treatment	Outcome
F1_P1	*IL12RB1*	c.1791+2T>G	M	7 m	26 y	Yes	BCG adenitis	Left axillary, cervical, abdomen	Yes	Yes	Nontyphoidal *Salmonella*	ATT for 6 m	Alive
F2_P2	*IL12RB1*	c.1095_1106del	M	5 m	11 m	Yes	BCG adenitis	Left axillary	-	-	-	ATT	Alive
F3_P3	*IL12RB1*	c.962C>A	F	6 m	-	Yes	BCG adenitis	Left axillary	-	-	-	ATT for 9 m	Dead
F4_P4	*IL12RB1*	c.962C>A	F	6 m	-	Yes	BCGosis	Left axillary, mandibular, abdomen	Yes	-	*Salmonella choleraesuis*	ATT twice for 9 m^a^	Dead
F5_P5	*IL12RB1*	c.962C>A	M	3 m	-	Yes	BCGosis	Left axillary, cervical, lungs, sepsis	Yes	-	*Candida *spp*.*	ATT	Dead
F6_P6	*IL12RB1*	c.1791+2T>G	M	3 m	-	No	BCG adenitis	Left axillary, submandibular, oral candidiasis	Yes	Yes	*Candida *spp*.*	ATT for 1 y	Lost to follow-up
F7_P7a	*IL12RB1*	c. 982delC/c. 982delC	M	2.4 m	25 y	Yes	BCGosis	Left axillary, lungs, IBD	Yes	-	*H. capsulatum*, *G. lamblia*	ATT thrice for 9 m, amphotericin B, i.v. antibiotics	Alive
F8_P8a	*IL12RB1*	c.1791+2T>G	F	6 m	-	Yes	BCG adenitis	Left axillary, cervical, and inguinal	-	-	-	ATT	Dead
F8_P8b	*IL12RB1*	c.1791+2T>G	F	3 m	7 y	Yes	BCG adenitis	Left axillary	-	-	-	ATT for 2 years	Alive
F9_P9	*IL12RB1*	c.962C>A	M	7 m	-	Yes	BCG adenitis	Left axillary, abdominal, cholangitis	-	-	-	ATT twice	Dead
F10_P10	*IL12RB1*	c.(1327+1_1328-1)_(1483+1_1484-1)del	M	5 m	5.5 y	No	BCG adenitis	Left axillary	-	-	-	ATT for 9 m	Alive
F11_P11	*IL12RB1*	c.962C>A	F	3 m	-	Yes	BCG adenitis	Left axillary	-	-	-	ATT for 9 m	Dead
F12_P12	*IL12RB1*	c.493C>T	M	6 y	-	Yes	No	Left axillary, cervical, skin, chest wall abscess	Yes	Yes	-	MDR ATT for 18 m, clarithromycin	Dead
F13_P13	*IL12RB1*	c.632G>C	F	4.5 m	-	Yes	BCGosis	Left axillary, GI tract, Skin	Yes	-	-	ATT for 9 m	Dead
F14_P14	*IL12RB1*	c.995G>A	M	10 m	-	NA	BCGosis	Left axillary, abdomen	Yes	-	-	First-line and second-line ATT	Dead
F15_P15	*IL12RB1*	c.587G>T	M	3 m	10.5 y	Yes	BCG adenitis	Left axillary, skin, liver, spleen	Yes	-	-	Sirolimus	Alive
F16_P16	*IL12RB1*	c.962C>A	M	3.6 m	15 y	NA	BCGosis	Left axillary, inguinal, cervical, abdominal, liver abscess, brain	Yes	-	-	Multiple ATT	Alive
F17_P17a	*IL12RB1*	c.599del	F	7 m	14 y	No	BCG adenitis	Left axillary, kidney	-	-	-	ATT	Alive
F18_P18	*IL12RB1*	c.1242C>G	M	4 m	-	Yes	BCGosis	Left axillary, cervical, and inguinal, liver, spleen	-	-	-	​	Dead
F19_P19	*IL12RB1*	c.1791+2T>G	M	4 m	13.5 y	Yes	BCG adenitis	Left axillary, perihilar, skin rash	-	-	*Salmonella enterica*	i.v. antibiotics	Alive
F7_P7b	*IL12RB1*	c. 982delC/c. 982delC	M	4 m	-	Yes	BCG adenitis	Left axillary, cervical, skin rash	-	-	-	ATT	Lost to follow-up
F17_P17b	*IL12RB1*	c.599del	F	7 m	19 y	No	BCGosis	Left axillary, cervical, liver, spleen, skin	-	-	-	First-line (6 m) and second-line (1 year) ATT, oral ATT (6 m)	Alive
F20_P20	*IL12RB1*	chr19:g.(18083492_18086759)_(18086933_18098754)del	F	3 m	4 y	No	BCGosis	Left axillary, cervical, and inguinal, liver, spleen	-	-	-	ATT	Alive
F21_P21	*IL12RB1*	c.1791+2T>G	M	3 m	2.5 y	No	BCG adenitis	Left axillary	-	Yes	-	ATT, i.v. antibiotics, IVIG	Alive
F22_P22a	*IL12RB1*	c.587G>T	M	1 y	-	NA	BCG adenitis	Left axillary	-	-	-	ATT	Lost to follow-up
F22_P22b	*IL12RB1*	c.587G>T	F	1 y	-	NA	BCG adenitis	Left axillary, inguinal	-	-	-	ATT	Lost to follow-up
F23_P23	*IL12RB1*	c.494del	M	5 y	14 y	Yes	BCG adenitis	Left axillary, cervical, mediastinal, inguinal, abdominal, retroperitoneal, lungs	Yes	-	Nontyphoidal *Salmonella*, *Nocardia *spp*.*	ATT, cephalosporin, fluconazole, IFN-α	Alive
F24_P24	*IL12RB1*	c.1189+1del	F	3 m	2 y	Yes	BCG adenitis	Left axillary	-	-	-	Amoxicillin, ATT 9 m	Alive
F25_P25	*IL12RB1*	c.515dup	M	3 m	-	NA	BCG adenitis	Left axillary, oral thrush	-	-	*Candida *spp*.*	ATT	Lost to follow-up
F26_P26	*IL12RB1*	c.962C>A	M	2 m	5 y	No	BCG adenitis	Left axillary, cervical, lungs	-	-	​	ATT	Alive
F44_P44	*IL12B*	c.429G>A	M	3 m	27 y	Yes	BCGosis	Liver, spleen	-	-	-	ATT thrice	Alive

F, Female; FTT, failure to thrive; IVIG, intravenous immunoglobulin; M, male; m, months; y, years; NA, not available.

a refers to patients who received two separate courses of ATT, each lasting 9 months.

Failure to thrive was observed in 11 patients (36%), and pneumonia occurred in seven (23%). Patient F12_P12, though lacking BCG complications, developed recurrent cervical lymphadenopathies. Mycobacterial infections were confirmed in eight patients via GeneXpert and in three patients by acid-fast bacilli (AFB) staining. Additional infections included disseminated nontyphoidal *Salmonella* in F19_P19 and F23_P23, and *Candida* spp. infection in F5_P5, F6_P6, and F25_P25. *Histoplasma capsulatum* and *Giardia lamblia* were isolated in one patient (F7_P7a).

Nine patients (F1_P1, F7_P7a, F9_P9, F12_P12, F14_P14, F16_P16, F17_P17a, F17_P17b, and F23_P23) developed disseminated tuberculosis later in life, with involvement of lymph nodes (cervical, inguinal, abdominal) and organs such as the lungs, skin (scrofuloderma), liver, kidneys, abdomen, and brain. Inflammatory bowel disease (IBD) was reported in three patients (F1_P1, F7_P7a, and F25_P25). Out of these nine patients, multidrug-resistant tuberculosis (MDR-TB) was identified in F14_P14, F17_P17a, and F17_P17b. Infection with *Mycobacterium ulcerans* was confirmed in F12_P12 by skin biopsy. In the remaining five patients, although culture confirmation was not available, the disseminated disease was likely representing a relapse of BCG infection.

### Immunological findings in patients with impaired IFN-γ production

Lymphocyte subset analysis was performed on 27 patients, of whom eight exhibited severe lymphopenia. The median absolute lymphocyte count among these patients was 836.5, with an interquartile range of 454.5–2,186.5. Among these, two patients (F17_P17a and F17_P17b) showed reduced naïve CD4^+^ (Th) and CD8^+^ (Tc) T cell subsets. Respiratory burst activity was normal in all patients tested.

**Figure 3.**
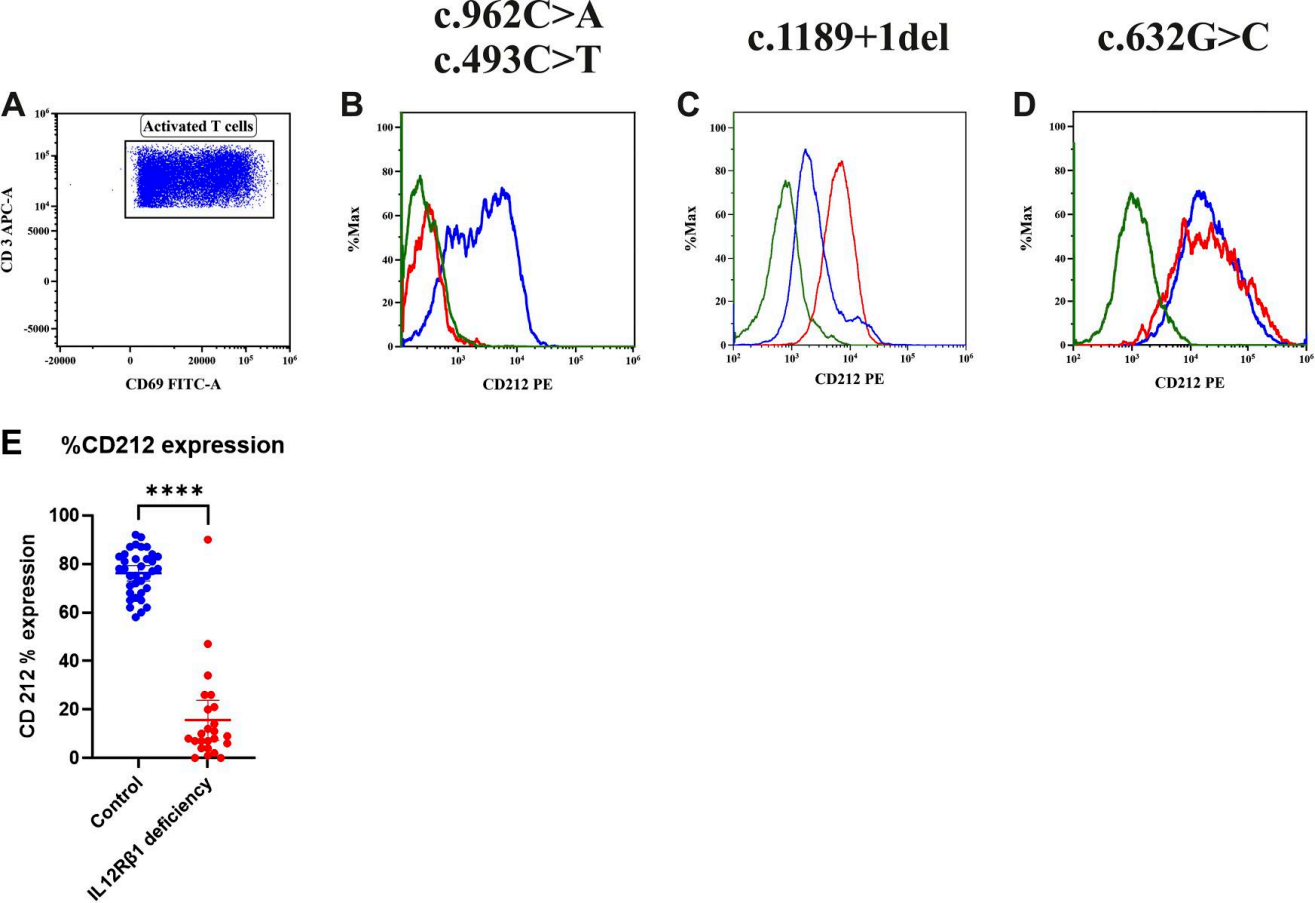
**Expression of CD212 on activated T cells in healthy controls and patients by flow cytometry. (A)** Gating of activated T cells using CD3 APC and CD69 FITC. **(B–D)** Patients with complete IL-12Rβ1 deficiency showing (B) absent, (C) reduced, and (D) normal expression of nonfunctional receptors on the surface of the cells in patients after stimulation with PHA and IL-2 compared with healthy controls. **(E)** Percent expression of CD212 in IL-12Rβ1 deficiency patients (Asterisks indicate statistical significance: ****P < 0.0001).

In patients with IL-12Rβ1 deficiency, the surface expression of CD212 showed considerable variability, correlating with the type and position of the underlying mutations. Patients with nonsense variants (*n* = 9) exhibited markedly reduced CD212 expression, ranging from 0% to 15%, while those with splice-site and frameshift variants (*n* = 7) showed expression levels between 4% and 35%. The missense variant c.587G>T, identified in three patients, was consistently associated with low CD212 expression (7%). Interestingly, patient P14, carrying a nonfunctional missense variant, displayed normal CD212 expression (90%) but impaired signaling, as indicated by a low pSTAT4 stimulation index (SI = 17), suggesting that surface expression does not always reflect receptor functionality (Fig. 3).

**Figure 4.**
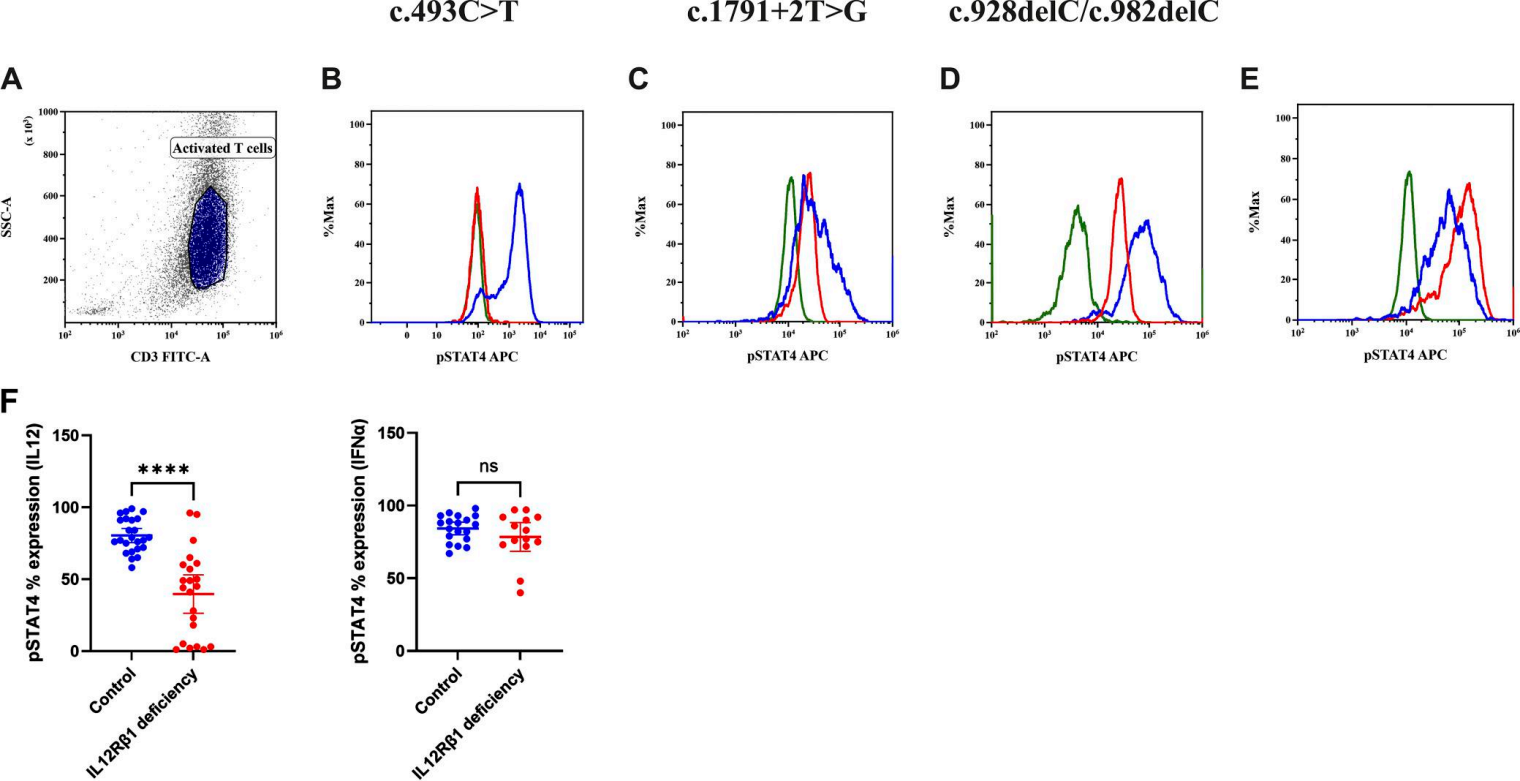
**Flow cytometric expression of pSTAT4 on activated T cells. (A)** Gating of activated T cells on side scatter versus CD3 FITC. **(B–D)** Complete IL-12Rβ1 deficiency showing (B) absent, (C) reduced expression, and (D) reduced MFI in patients after stimulation with IL-12p70 compared with healthy controls, where green is patient unstained, red patient stained, and blue control stained. **(E)** Normal pSTAT4 expression in both IL-12Rβ1–deficient patients and controls after stimulation with IFN-α. **(F)** Percent expression of pSTAT4 in IL-12Rβ1 deficiency patients vs. controls after stimulation with IL-12p70 (asterisks indicate statistical significance: ****P < 0.0001) and IFN-α (ns: not significant).

Among patients with nonsense variants, pSTAT4 responses to IL-12p70 stimulation varied widely (0–96%, SI 0.5–7), whereas responses to IFN-α remained intact in all cases (Fig. 4).

**Figure 5.**
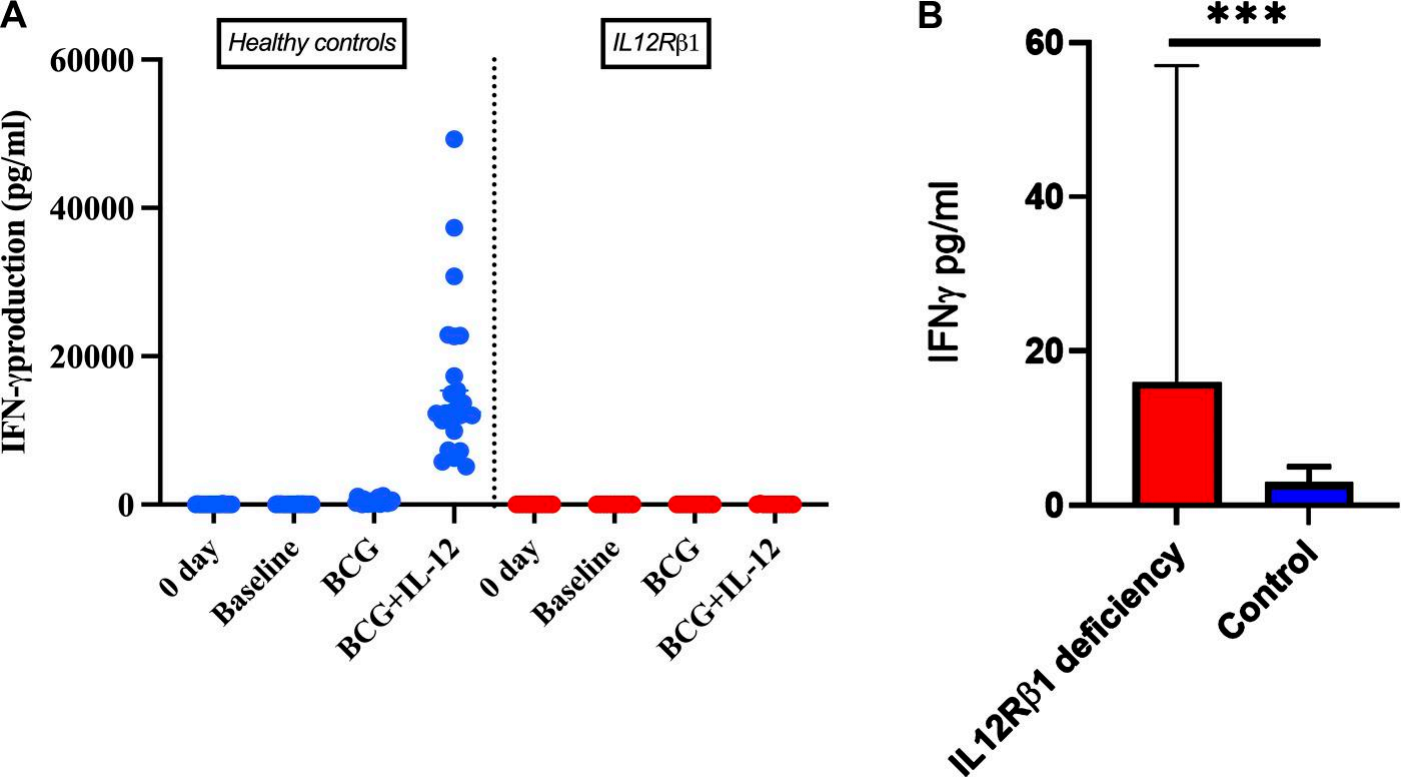
**Defective IFN-γ production following IL-12 stimulation and altered baseline IFN-γ levels in IL-12Rβ1–deficient patients. (A)** Impaired cellular response to IL-12p70. Production of IFN-γ by whole blood from IL-12Rβ1–deficient patients and controls, either unstimulated or stimulated with BCG alone or with BCG and recombinant IL-12p70. **(B)** Baseline serum IFN-γ levels in patients with IL-12Rβ1 deficiency and controls (asterisks indicate statistical significance: ***P < 0.0001).

Functional cytokine assays in 13 patients with IL-12Rβ1 deficiency confirmed severely impaired IFN-γ production, ranging from 1 to 147 pg/ml (Fig. 5 A).

Additionally, baseline serum IFN-γ levels measured in 22 patients ranged from 1 to 900 pg/ml (Fig. 5 B), reflecting defective IL-12 signaling and downstream immune activation. IFN-γ release assay (IGRA) results were indeterminate or negative in three patients, reflecting possible impaired IFN-γ production consistent with IL-12Rβ1 deficiency (Table 2).

**Table 2.** IGRA results in patients with IL-12Rβ1 deficiency

Patient	Gene	Nucleotide change	TB1-Nil	TB2-Nil	Mitogen-Nil	IGRA result
F2_P2	*IL12RB1*	c.1095_1106del	0	0	0	Indeterminate
F16_P16	*IL12RB1*	c.962C>A	0	0	0.95	Negative
F20_P20	*IL12RB1*	chr19:g.(18083492_18086759)_(18086933_18098754)del	0.1	0.1	0.5	Negative

TB, tuberculosis.

### Clinical and microbiological findings in patients with impaired response to IFN-γ

Among the 50 molecularly characterized MSMD patients, 19 demonstrated impaired cellular responses to IFN-γ. These included 10 patients with IFN-γR1 deficiency (F27_P27-F36_P36), six with AR complete IFN-γR2 deficiency (F37_P37a-F41_P41), and three with AR complete STAT1 deficiency (F42_P42a-F43_P43). All patients with an impaired cellular response to IFN-γ had an early disease onset, with a median age at presentation of 2 mo (Table 3).

**Table 3.** Clinical and microbiological findings in patients with impaired response to IFN-γ

Patient	Sex	Age at onset	Current age	Consanguinity	BCG infection	Other organ involvement	FTT	Diarrhea	Other organism	Treatment	Outcome
F27_P27	F	2.4 m	-	Yes	BCGosis	Liver, spleen	-	-	CMV	Solonex, rifampicin, ofloxacin, ethambutol, Valgan 50 mg	Dead
F28_P28	F	2 m	-	Yes	BCGosis	Liver, spleen	-	-	-	ATT	Dead
F29_P29	M	1 m	10 y	Yes	BCGosis	Lymph node, bone, skin, meninges, lungs, liver, spleen	-	Yes	-	Prednisolone, ATT	Alive
F30_P30	M	6 m	-	Yes	BCGosis	Liver, spleen, skin	-	-	-	ATT	Dead
F31_P31	F	1 m	-	Yes	BCGosis	Liver, spleen, lungs, skin	Yes	-	*Enterobacter *spp*.*, *C. guilliermondii*	ATT	Dead
F32_P32	M	1 m	-	No	No	Abdomen	-	-	-	-	Dead
F33_P33	F	2 m	10.5 y	No	BCGosis	Liver, spleen, bone	-	-	CMV, *P. aeruginosa*	ATT	Alive
F34_P34	F	1 m	11 y	No	BCGosis	Bone	-	-	-	ATT	Alive
F35_P35	F	3 m	6 y	No	BCGosis	Liver, spleen	-	-	-	ATT	Alive
F36_P36	F	3.5 y	17 y	No	BCGosis	Skin, bone, liver, spleen	-	-	*M. abscessus*, *Staphylococcus aureus*, *M. contagiosum*	IFN-α, ATT, antibiotics	Alive
F37_P37a	M	6 m	-	Yes	Not vaccinated	Lungs, abdomen, liver, spleen	-	-	CMV	Valganciclovir, ATT	Died 2 years later with unrelated cause
F37_P37b	M	9 m	14 y	Yes	Not vaccinated	Lungs	-	-	CMV, *Neisseria *spp*.*	Valganciclovir, ATT, antibiotics	Transplanted, alive
F38_P38	M	3 m	-	Yes	BCGosis	Lungs	-	Yes	-	ATT	Lost to follow-up
F39_P39	M	2 m	-	Yes	BCGosis	Lungs, abdomen, liver, spleen	-	-	-	ATT	Dead
F40_P40	M	2 m	6 y	Yes	BCGosis	Liver, spleen	-	-	-	ATT	Alive
F41_P41	M	3 m	2 y	Yes	BCGosis	Liver, spleen	-	-	HSV	ATT, clarithromycin, acyclovir	Alive
F42_P42a	F	2 m	-	Yes	BCG adenitis	Lungs	Yes	-	-	Rifampicin, ethambutol, isoniazid, Septran	Dead
F42_P42b	F	3 m	-	Yes	BCG adenitis	-	-	-	-	ATT	Dead
F43_P43	M	2.4 m	-	Yes	BCG adenitis	-	-	-	*Acinetobacter *spp*.*, *Klebsiella *spp*.*, CMV	ATT, Taxim, valganciclovir	Dead

F, female; FTT, failure to thrive; M, male; m, months; y, years.

### IFN-γR1 deficiency

A total of 10 patients in our cohort were identified with IFN-γR1 deficiency. Patients with IFN-γR1 deficiency exhibited an early onset of symptoms, with the median age at presentation being just 2 mo. Consanguinity was observed in five families, all of which had a complete autosomal recessive deficiency (F27_P27–F31_P31). Sibling death was noted in three families. The patients presented with disseminated BCG infections affecting multiple organs, including the lymph nodes, liver, spleen, skin, and lungs. Histopathology and microbiological testing (AFB staining, culture, and GeneXpert) supported the diagnosis of mycobacterial infection in four cases. Additional infections included cytomegalovirus (CMV), *Candida guilliermondii*, and *Enterobacter* spp.

In contrast, consanguinity was absent in four families with partial dominant (PD) IFN-γR1 deficiencies (F33_P33-F36_P36). These patients presented with BCGosis, affecting the lymph nodes, liver, spleen, and bones. Osteomyelitis due to mycobacterial infections was a common clinical manifestation seen in those with PD deficiencies. One patient (F36_P36) had BCGosis, with the second episode confirmed as *Mycobacteroides abscessus* infection. One patient (F33_P33) had infections with *MTB* complex, CMV, and *Pseudomonas aeruginosa*. Nonmycobacterial presentations included autoimmune hemolytic anemia (AIHA), abdominal distension with hepatic microabscesses, and pericardial effusion.

### Immunological findings

**Figure 6.**
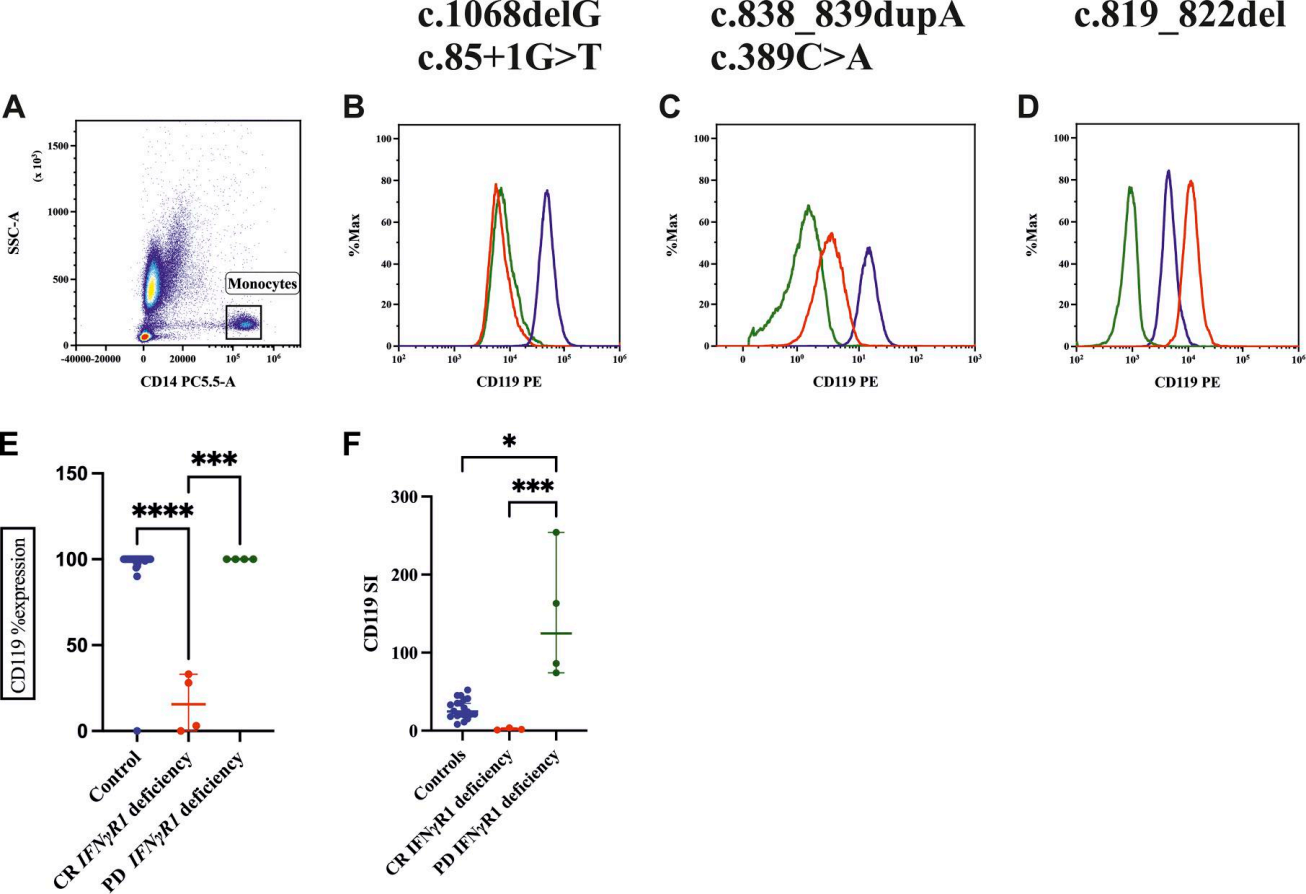
**Flow cytometric assessment of CD119 expression on monocytes. **Overlay of patients with CR and PD forms of IFN-γR1 deficiencies. **(A)** Gating of monocytes on side scatter vs. CD14 PCP5.5. **(B)** CR deficiency showing absent expression of CD119 where green is patient unstained, red patient stained, and blue control stained. **(C)** CR deficiency showing reduced expression compared with a healthy control. **(D)** PD deficiency showing increased expression. **(E)** Percent expression, controls vs. CR IFN-γR1 deficiencies (asterisks indicate statistical significance: ****P < 0.0001), CR IFN-γR1 deficiencies vs. PD IFN-γR1 deficiencies (asterisks indicate statistical significance: ***P < 0.0001). **(F)** SI of controls vs. PD IFN-γR1 deficiency (asterisks indicate statistical significance: *P < 0.0172), CR IFN-γR1 deficiencies vs. PD IFN-γR1 deficiencies (asterisks indicate statistical significance: ***P < 0.0003).

Lymphocyte subset analysis was conducted on all patients with IFN-γR1 deficiency. Patient F36_P36 exhibited severe lymphopenia with low Th and Tc naïve cells. All patients with IFN-γR1 deficiency exhibited normal respiratory bursts. Out of the 50, CD119 surface expression was analyzed in 34 patients. Of these, 26 patients (88%) with IL-12Rβ1, IFN-γR2, and STAT1 defects showed normal receptor expression (95–100%), comparable to healthy controls. Four patients with PD IFN-γR1 deficiency exhibited elevated median fluorescence intensity (MFI), with SI of 254 (F33_P33), 163 (F34_P34), 86 (F35_P35), and 74 (F36_P36). In contrast, among the four patients with complete recessive (CR) IFN-γR1 deficiency, two (F27_P27 and F28_P28) showed reduced receptor expression (20–30%), while two (F29_P29 and F31_P31) had complete absence of expression (0–3%; Fig. 6).

**Figure 7.**
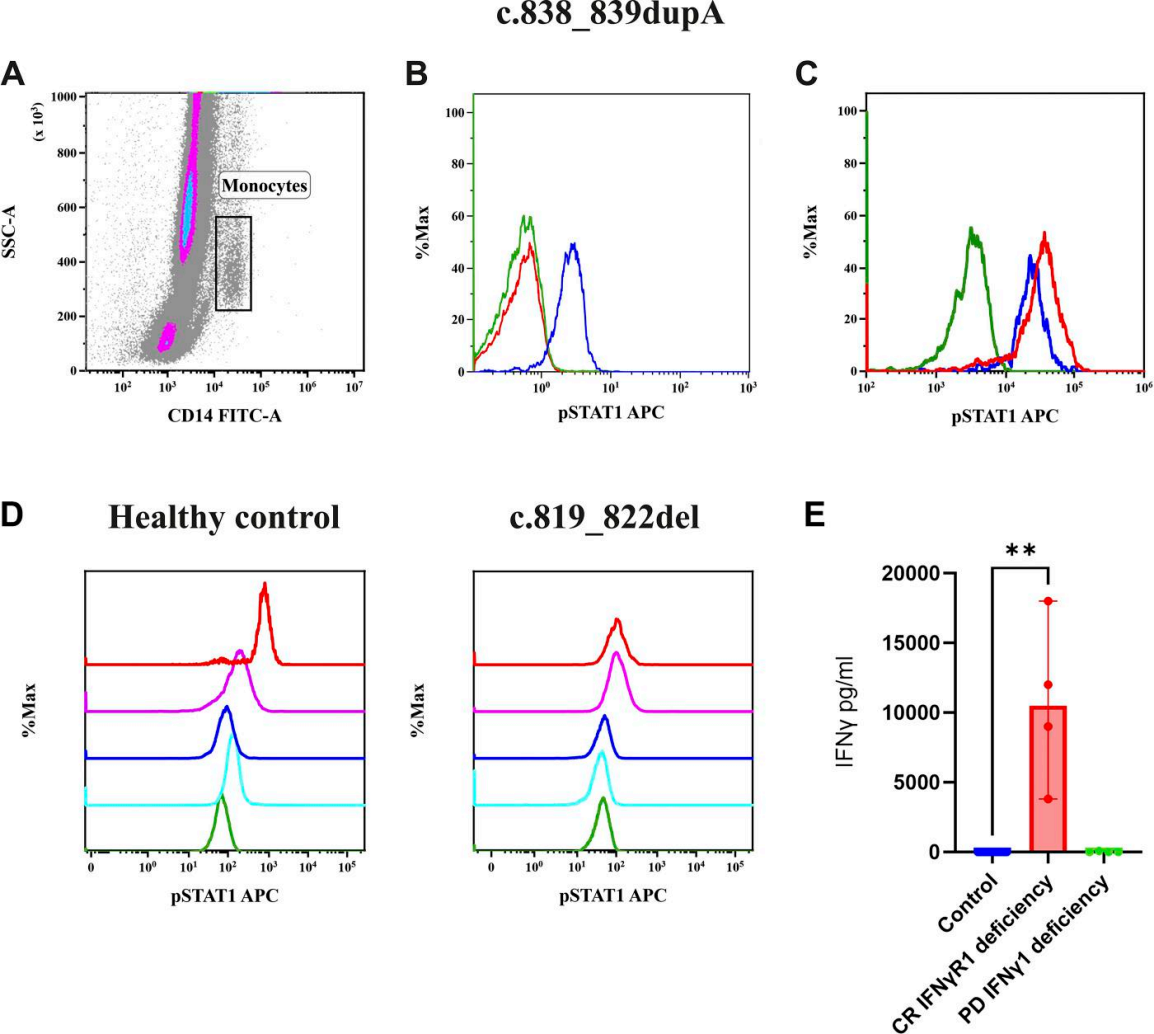
**Flow cytometric assessment of pSTAT1 expression on monocytes.** **(A)** Gating of monocytes on side scatter vs. CD14 FITC. **(B)** Overlay of patients with CR IFN-γR1 deficiencies showing an abolished response to IFN-γ. **(C)** Patients with CR IFN-γR1 showing a normal response to IFN-α compared with healthy controls, where green is patient unstained, red patient stained, and blue control stained. **(D)** Overlay of pSTAT1 expression in a healthy control and patient with PD IFN-γR1 deficiency showing an impaired response to increasing concentrations of IFN-γ (green is unstained, sky blue 10 IU/ml, violet 100 IU/ml, magenta 1,000 IU/ml, and red 10,000 IU/ml concentration of IFN-γ). **(E)** Baseline serum IFN-γ levels in patients with complete and partial IFN-γR1 deficiency, and controls (asterisks indicate statistical significance: **P < 0.0014).

**Figure S1.**
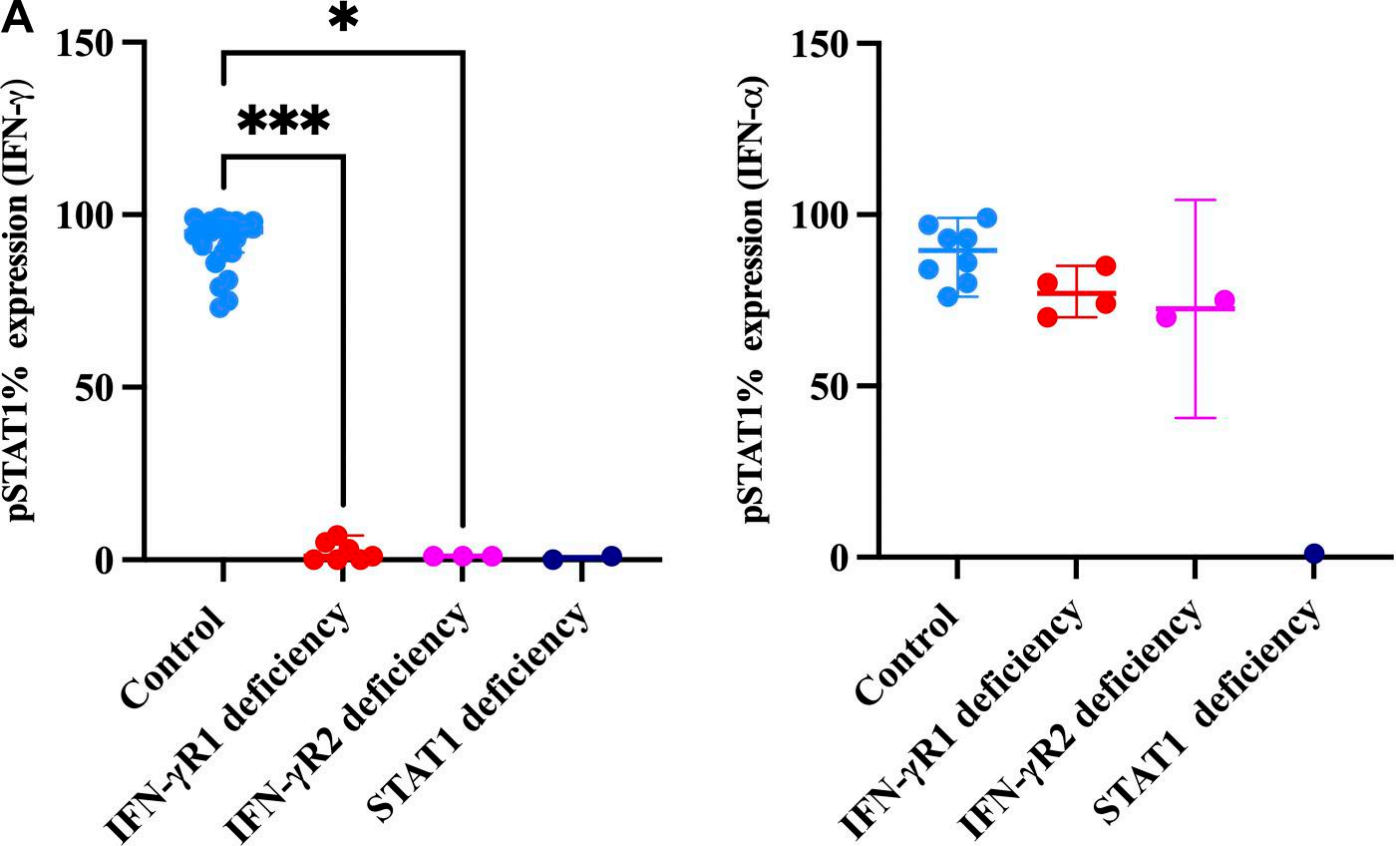
**Impaired STAT1 phosphorylation in patients with impaired response to IFN-γ relative to healthy controls. (A) **Percent expression of pSTAT1 in controls vs. IFN-γR1 deficiencies (asterisks indicate statistical significance: ***P < 0.0006) and controls vs. IFN-γR2 deficiencies (asterisks indicate statistical significance: *P < 0.0402).

Patients with CR IFN-γR1 deficiency demonstrated a complete lack of STAT1 phosphorylation in response to IFN-γ stimulation, while pSTAT1 induction remained normal with IFN-α, confirming pathway specificity (Fig. 7). In contrast, patients with PD IFN-γR1 deficiency exhibited a partial response to increasing concentrations of IFN-γ, indicating impaired but not absent signaling (Fig. 7 D). Two patients with IFN-γR1 deficiency showed absent IL-12p70 production upon IFN-γ stimulation, indicating a defective cellular response (Fig. S1). Baseline serum IFN-γ levels were markedly elevated in patients with CR deficiencies, ranging from 150 to 20,000 pg/ml, while patients with PD deficiencies had moderately elevated levels, ranging from 5 to 80 pg/ml (Fig. 7 E).

### IFN-γR2 deficiency

In total, six patients from five families in our cohort were presented with IFN-γR2 deficiency. The median age at onset of symptoms was 3 mo. The median age of diagnosis was 9 mo. All families in the cohort showed signs of consanguinity. In our cohort, four patients with IFN-γR2 deficiency presented soon after receiving the BCG vaccination. They exhibited symptoms such as left axillary lymphadenitis, cervical lymphadenopathy, pneumonia, hepatosplenomegaly, and diarrhea. Two siblings from the same family did not receive the BCG vaccine due to a history of early death in the family linked to BCG-related complications. These siblings presented with cervical lymphadenopathy and pneumonia. Additionally, one sibling (F37_P37a) developed dry, scaly skin. Along with mycobacterial infections, three patients (F37_P37a, F37_P37b, and F41_P41) also experienced viral infections, including CMV and HSV.

### Immunological findings

**Figure 8.**
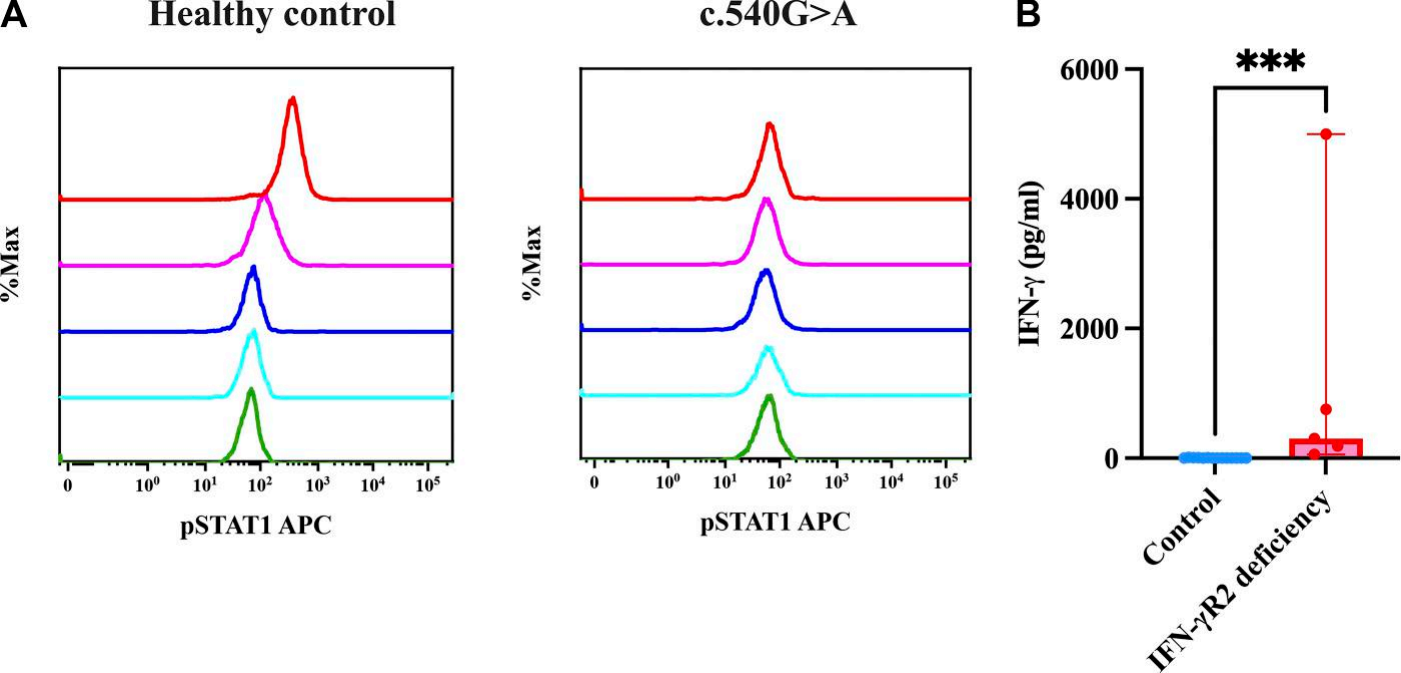
**Abolished STAT1 phosphorylation in response to IFN-γ and altered baseline serum IFN-γ levels in patients with complete IFN-γR2 deficiency. (A)** Overlay of pSTAT1 expression in a healthy control and patient with CR IFN-γR2 deficiency showing an abolished response to increasing concentrations of IFN-γ (green is unstained, sky blue 10 IU/ml, violet 100 IU/ml, magenta 1,000 IU/ml, and red 10,000 IU/ml concentration of IFN-γ). **(B)** Baseline serum IFN-γ levels of patients with complete IFN-γR2 deficiency and controls (Asterisks indicate statistical significance: P 0.0002***).

**Figure S2.**
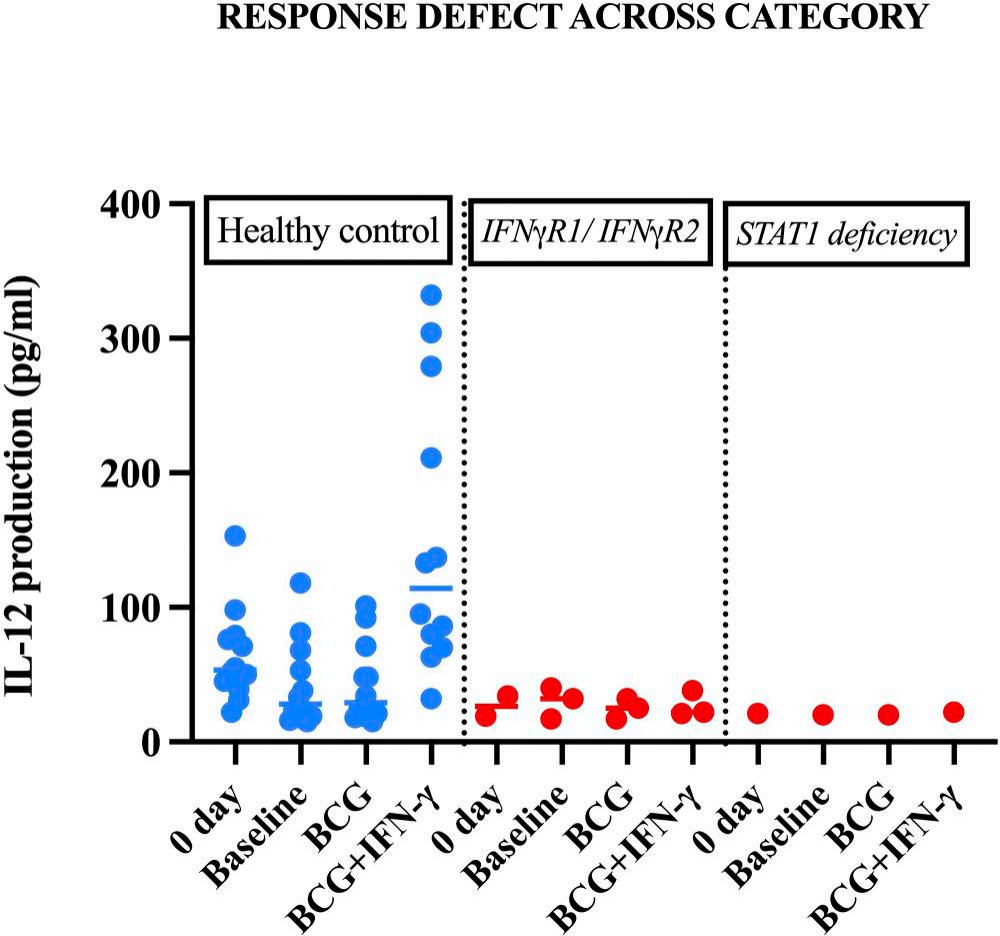
**Impaired cellular response to IFN-γ.** Production of IL-12p70 by whole blood from IFN-γR1–, IFN-γR2–, and STAT1-deficient patients and controls, either unstimulated or stimulated with BCG alone or with BCG and recombinant IFN-γ.

None of the patients exhibited STAT1 phosphorylation upon IFN-γ stimulation; however, they did respond to IFN-α (Fig. 8 A and Fig. S2). A defect in IL-12p70 production was observed in response to IFN-γ (Fig. S1), although IFN-γ production in response to IL-12p70 remained normal. Additionally, four patients (F37_P37a, F37_P37b, F38_P38, and F40_P40) showed elevated baseline levels of IFN-γ, ranging from 150 to 5,000 pg/ml (Fig. 8 B).

### STAT1 deficiency

Patients with this deficiency presented early, before 3 mo of age, with severe complications. In one consanguineous family, two siblings (F42_P42a and F42_P42b) developed BCG adenitis around 3 mo of age, with confirmed AFB positivity and required antitubercular therapy (ATT). Another male patient (F43_P43), also from a consanguineous background, developed BCG adenitis at 2 mo, along with bacterial (*Acinetobacter*, *Klebsiella*) and viral (CMV) coinfections. Despite appropriate treatment, all three patients succumbed to their illness.

### Immunological findings

**Figure 9.**
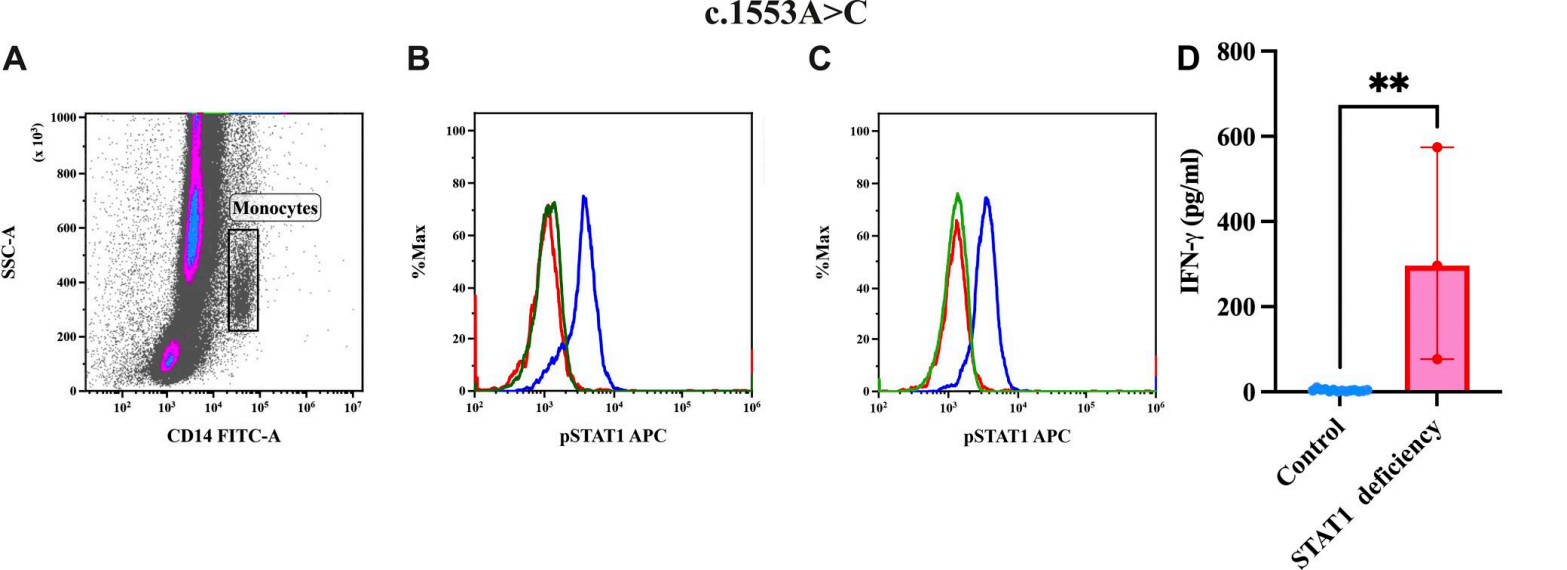
**Flow cytometric assessment of pSTAT1 expression on monocytes.** **(A)** Gating of monocytes on side scatter vs. CD14 FITC. **(B)** Overlay of a patient with complete STAT1 deficiency shows an abolished response to both IFN-γ compared with a healthy control. **(C)** Overlay of a patient with complete STAT1 deficiency shows an abolished response to IFN-α compared with a healthy control, where green is patient unstained, red patient stained, and blue control stained. **(D)** Baseline serum IFN-γ levels in patients with STAT1 deficiency and controls (Asterisks indicate statistical significance: P 0.0015**).

STAT1 phosphorylation was abolished in patients upon stimulation with both IFN-γ and IFN-α (Fig. 9 and Fig. S1). IFN-γ production was normal in response to IL-12p70, but there was no increase in IL-12p70 production in response to BCG+IFN-γ (Fig. S2). Two patients displayed elevated IFN-γ levels in the range of 250–600 pg/ml (Fig. 9 D).

## Discussion

We present a comprehensive analysis of a large cohort of 50 molecularly confirmed MSMD patients, including detailed functional evaluations. BCG-related complications emerged as the most frequent infectious manifestation in this cohort. BCG complications can present as a localized BCG adenitis, which was observed in nearly 48% of the MSMD patients in our cohort, all of whom required ATT. Although not all patients with localized BCG disease have an underlying immunodeficiency, our previous study showed that nearly 58% of them had an inborn error of immunity (IEI), with MSMD being the most common (12). Therefore, in countries where BCG vaccination is given at birth, even localized BCG adenitis needs to be considered as a warning sign for IEI, especially if it requires therapeutic intervention.

The severity and outcomes of BCG-related complications differed markedly among the genetic groups. Patients with complete IFN-γR1 and IFN-γR2 deficiencies had the most severe outcomes, with high rates of disseminated BCGosis and mortality, consistent with the critical role of IFN-γ signaling in mycobacterial control. Conversely, patients with PD IFN-γR1 deficiency had milder phenotypes, surviving without significant BCG complications, highlighting a genotype–phenotype correlation. IL-12Rβ1–deficient patients showed variable severity, with some developing severe complications but others experiencing only localized adenitis or no complications, suggesting heterogeneity in immune impairment. Interestingly, all patients with STAT1 deficiency died despite only having BCG adenitis, indicating that even localized disease in this group can be life-threatening and may reflect broader susceptibility to infection.

Previous studies have reported that MSMD patients typically do not develop secondary mycobacterial infections after an initial diagnosis of BCG disease (13). However, in our cohort, 10 patients experienced recurrent tuberculosis (14); however, molecular confirmation was available only for three patients, all of whom were confirmed to have MDR-TB. In the remaining cases, reinfection with *MTB* complex or other mycobacteria could not be confirmed microbiologically.

In our cohort, nontuberculous mycobacterial (NTM) infections, commonly reported as the predominant clinical presentation in TB nonendemic countries, were identified in only two patients. This likely reflects underreporting. In India, where the TB burden is high, AFB-positive cases are often presumed to be TB and are empirically treated with standard anti-TB therapy. Although molecular assays like Cartridge-Based Nucleic Acid Amplification Test or TrueNAT are now widely available through the National Tuberculosis Elimination Program, these tests do not detect NTM infections. Furthermore, access to Mycobacteria Growth Indicator Tube culture and subsequent molecular testing essential for confirming NTM species is limited. Given that treatment regimen differs significantly between MTB and NTM infections, early and accurate species identification, coupled with antimicrobial susceptibility testing, is imperative in patients with MSMD and should be prioritized.

Mucocutaneous *Candida* infections are frequently seen in patients with IL-12Rβ1 deficiency but have not been reported in those with IFN-γR1 deficiency. Notably, *C. guilliermondii* was isolated from patient F31_P31, who was diagnosed with IFN-γR1 deficiency. Supporting this finding, studies in IFN-γR1 knockout mice have demonstrated an essential role of IFN-γ in the clearance of *Candida*, with deficient mice exhibiting increased susceptibility to infection (15).

Six patients with IL-12Rβ1 deficiency presented with diarrhea or other gastrointestinal manifestations. Among them, three patients were diagnosed with IBD by gastroenterologists, based on colonoscopic findings and histopathological examination. In the remaining patients, a definitive diagnosis could not be established. Their symptoms may be of infectious origin potentially due to pathogens such as *Salmonella* or mycobacteria, which are common in MSMD or may represent early signs of immune dysregulation, including the potential development of IBD. Given that IL-12Rβ1 deficiency is a known monogenic cause associated with IBD, it remains possible that these patients could develop IBD later in the disease course.

The role of IFN-γ in systemic autoimmunity is not yet fully understood. However, it is known to be important for T cell differentiation and immunoglobulin class switching in B cells. These functions suggest that IFN-γ plays a key role in the immune responses that drive autoimmune diseases (16). In our cohort, patient F32_P32 with IFN-γR1 deficiency developed AIHA. This has not been reported before and raises questions about other pathways that may cause autoimmunity when IFN-γ signaling is absent.

MSMD is generally not considered in the differential diagnosis of lymphopenia. However, in our cohort, nine patients exhibited severe lymphopenia along with a reduction in naïve T cell subsets. Of these, three patients (F17_P17a, F17_P17b, and F36_P36) remained on ATT without any improvement in lymphocyte counts. In contrast, patient F12_P12 showed recovery of lymphocyte counts after discontinuing ATT. Interestingly, patient F7_P7a had persistent lymphopenia and reduced naïve T cells despite not receiving any treatment. No additional pathogenic variants were detected by NGS, and the cause of lymphopenia in this case remains unexplained.

*IL12RB1* variants accounted for 60% of molecularly characterized cases, confirming that IL-12Rβ1 deficiency is the most common genetic cause of MSMD globally, consistent with previous studies. The c.1791+2T>G splice-site variant in the *IL12RB1* gene is one of the most frequently reported variants in IL-12Rβ1 deficiency (17). However, this variant was identified in only six patients in our cohort. The nonsense variant c.962C>A was the most frequently observed mutation in our cohort, identified in seven patients. This variant has been associated with severe clinical outcomes; five patients died, and the remaining two continue to experience recurrent infections.

Patient F13_P13, who carried the previously reported R211P missense variant (18), demonstrated normal surface expression of CD212, in contrast to earlier reports where the same variant was associated with absent receptor expression. Despite normal expression, pSTAT4 signaling was impaired in our patient, indicating defective cytokine binding or downstream signaling. Previously reported case with this variant presented with extraintestinal Salmonellosis and had milder clinical courses. In contrast, our patient presented with gastroenteritis without *Salmonella* isolation and had a severe clinical outcome, ultimately resulting in death.

Patient F29_P29, diagnosed with CR IFN-γR1 deficiency, underwent two unsuccessful HSCT. The first attempt failed due to graft failure with subsequent autologous reconstitution, while the second was complicated by graft-versus-host disease (GVHD). Elevated IFN-γ levels may have played a role in triggering GVHD, underscoring the importance of pretransplant IFN-γ level assessment as a potential predictive marker for HSCT-related complications. The patient remains clinically stable on prednisolone and ATT but continues to show poor nutritional recovery. Interestingly, this patient also developed osteomyelitis, a manifestation typically associated with PD *IFN-γR1* deficiency and rarely observed in complete deficiency due to the high mortality in such cases. IFN-γR1 is expressed on osteoclasts, which are typically inhibited by IFN-γ. In patients with impaired IFN-γ signaling, this inhibition is disrupted. To our knowledge, this represents the first documented case of osteomyelitis in a patient with CR IFN-γR1 deficiency within our cohort.

In our cohort, 40% of patients succumbed to the disease, reflecting the high morbidity and mortality associated with MSMD. This mortality rate underscores the urgent need for early identification and consideration of HSCT in these patients.

Both functional and genetic approaches are available for studying the IFN-γ/IL-12-23 circuit. The application of these assays in both clinical and research settings has been well documented in the literature (10). These assays demonstrated consistent correlations, whether performed before or following molecular confirmation of genetic defects in our cohort. A correlation was observed between the severity of disease and protein expression in patients with IFN-γR1 deficiency: individuals with CR deficiency, characterized by absent or markedly reduced CD119 expression, presented with more severe clinical phenotypes compared to those with PD deficiency, who showed increased CD119 expression. Additionally, higher levels of circulating IFN-γ were noted in patients with CR deficiency. However, cytokine production assays did not show severity-related differences. In contrast, for IL-12Rβ1 deficiency, no correlation was observed between protein expression, cytokine data, and clinical severity, as even patients with normal protein expression exhibited severe disease manifestations.

Negative and indeterminate results in the IGRA, particularly with a low response in the mitogen (positive control) tube, may indicate impaired IFN-γ production. This finding can serve as a valuable screening tool for patients with impaired IFN-γ production, where access to specific laboratory investigations is not available. However, it needs to be tested in a larger number of patients.

In conclusion, BCG-related complications were the most common clinical manifestation in our cohort, highlighting the need for early screening for MSMD in patients who present with adverse reactions following BCG vaccination. Prompt evaluation can significantly reduce diagnostic delays and help prevent morbidity and mortality in these cases. In patients with a strong family history of early sibling deaths due to BCG complications, it is advisable to postpone BCG vaccination until immunological investigations are completed. It is also important to note that BCG-related complications are not exclusive to MSMD and may occur in other IEIs; hence, a broad differential diagnosis must be considered before proceeding with MSMD-specific evaluations. The use of flow cytometry and ELISA-based functional assays, in combination with molecular genetic analysis, enables the accurate identification of underlying genetic defects associated with MSMD. These diagnostic tools are instrumental in differentiating between complete and partial deficiencies in the IFN-γ/IL-12 axis. Such stratification is critical for tailoring patient management strategies depending on the underlying genetic defect.

## Materials and methods

### Study recruitment and ethics

Patients with genetically confirmed MSMD, referred to the Indian Council of Medical Research National Institute of Immunohematology (NIIH), were included in the study. The ethics committee of the institute approved this study (No. NIIH/IEC/02-2014). Written informed consent was obtained from all participants or their legal guardians prior to inclusion in the study. Following ethical approval and consent, 6–8 ml of blood was collected in EDTA, heparin, and plain vacutainer. A detailed clinical proforma was filled out for all patients.

### Immunological evaluation

Complete blood count was obtained for all the patients using the Sysmex XS-800i cell counter. For lymphocyte subset evaluation, samples were acquired on a flow cytometer. Analysis was done using Kaluza v2.1 software. The NBT test was performed on all patients using NBT dye (Catalog no. N 6876; Sigma-Aldrich), and the slides were examined under an oil immersion lens.

Flow cytometric analysis of IFN-γR1 expression on monocytes was performed using CD14 PerCP (A07765; Beckman Coulter) and CD119 PE (12-1199-42, GIR-208; eBiosciences) antibodies. IL-12Rβ1 expression on activated T cells was evaluated using CD3 APC (555342; Beckman Coulter), CD69 FITC (555530; BD), and CD212 PE (556065; BD) antibodies. Flow cytometric evaluation of pSTAT1 expression was performed via intracellular staining with pSTAT1 APC (612597; BD) following stimulation with hrIFN-γ (PHC4031; Gibco) and hrIFN-α (NBP2-26551; Novus), using CD14 FITC (347497; BD) for monocyte identification. Similarly, pSTAT4 expression was assessed in activated T cells using pSTAT4 APC (558137; BD) after stimulation with hrIL-12 (4161-10; BioVision) and hrIFN-α (NBP2-26551; Novus), with CD3 FITC (BD) used as a surface marker. Data were acquired on a flow cytometer and analyzed using Kaluza v2.1 software. The stain index was calculated.

IFN-γ and IL-12p70 production was measured after stimulation with live BCG, with or without costimulation by hrIL-12p70 (20 ng/ml) or hrIFN-γ (5,000 IU/ml). Baseline serum IFN-γ levels were measured using a BD OptEIA Human IFN-γ ELISA kit.

### Statistical analysis

Statistical analyses were performed to assess the significance of differences between patient groups. Specifically, comparisons between complete recessive and PD IFN-γR1 deficiencies were conducted using appropriate statistical tests. P values <0.05 were considered statistically significant. GraphPad Prism 10 software was used to perform statistical analysis.

### Molecular analysis

DNA was extracted from both fresh and stored blood samples using commercially available spin column–based nucleic acid purification kits (Qiagen). DNA concentration was measured using the Qubit fluorometer. Sanger sequencing was employed to analyze frequently mutated genes such as *IL12RB1* and *IFNGR1* in 11 patients. For the remaining patients, NGS was performed. Parental segregation analysis was conducted for families based on the availability of parental samples. Whole-exome sequencing was conducted by MedGenome following the submission of samples for NGS.

### Online supplemental material

The supplementary material includes two figures and one table. Fig. S1 shows the statistically significant difference in pSTAT1 expression after stimulation with IFN-γ and IFN-α between controls and MSMD patients with different deficiencies. Fig. S2 shows IL-12p70 production in controls and patients with MSMD. Table S1 shows the molecular and functional characterization of 50 MSMD patients.

## Supplementary Material

Table S1 shows the molecular and functional characterization of 50 MSMD patients.

## Data Availability

The data underlying this study are not publicly available due to patient privacy issues. The data are available from the corresponding author upon request.
